# Bioluminescence Resonance Energy Transfer Based G Protein-Activation Assay to Probe Duration of Antagonism at the Histamine H_3_ Receptor

**DOI:** 10.3390/ijms20153724

**Published:** 2019-07-30

**Authors:** Tamara A. M. Mocking, Maurice C. M. L. Buzink, Rob Leurs, Henry F. Vischer

**Affiliations:** Amsterdam Institute for Molecules, Medicines and Systems (AIMMS), Division of Medicinal Chemistry, Faculty of Science, Vrije Universiteit Amsterdam, De Boelelaan 1108, 1081 HZ Amsterdam, The Netherlands

**Keywords:** histamine H_3_ receptor (H_3_R), G protein-coupled receptor (GPCR), re-equilibration, ligand binding kinetics, residence time

## Abstract

Duration of receptor antagonism, measured as the recovery of agonist responsiveness, is gaining attention as a method to evaluate the ‘effective’ target-residence for antagonists. These functional assays might be a good alternative for kinetic binding assays in competition with radiolabeled or fluorescent ligands, as they are performed on intact cells and better reflect consequences of dynamic cellular processes on duration of receptor antagonism. Here, we used a bioluminescence resonance energy transfer (BRET)-based assay that monitors heterotrimeric G protein activation via scavenging of released Venus-Gβ_1_γ_2_ by NanoLuc (Nluc)-tagged membrane-associated-C-terminal fragment of G protein-coupled receptor kinase 3 (masGRK3ct-Nluc) as a tool to probe duration of G protein-coupled receptor (GPCR) antagonism. The Gα_i_-coupled histamine H_3_ receptor (H_3_R) was used in this study as prolonged antagonism is associated with adverse events (e.g., insomnia) and consequently, short-residence time ligands might be preferred. Due to its fast and prolonged response, this assay can be used to determine the duration of functional antagonism by measuring the recovery of agonist responsiveness upon washout of pre-bound antagonist, and to assess antagonist re-equilibration time via Schild-plot analysis. Re-equilibration of pre-incubated antagonist with agonist and receptor could be followed in time to monitor the transition from insurmountable to surmountable antagonism. The BRET-based G protein activation assay can detect differences in the recovery of H_3_R responsiveness and re-equilibration of pre-bound antagonists between the tested H_3_R antagonists. Fast dissociation kinetics were observed for marketed drug pitolisant (Wakix^®^) in this assay, which suggests that short residence time might be beneficial for therapeutic targeting of the H_3_R.

## 1. Introduction

Drug-target binding kinetics has established its relevance in drug discovery as target residence time of a drug might better predict its in vivo effect, as compared to its equilibrium binding affinity (pK_i_) for the target [1]. Residence time is the reciprocal of the dissociation rate constant (*k_off_*) of a receptor-bound drug, which has mainly been derived from competition binding assays using the Motulsky and Mahan method [2]. Recently, a bioluminescent resonance energy transfer (BRET)-based binding assay on living cells using a Nanoluc (Nluc)-tagged receptor and fluorescent tracer has been introduced for determination of kinetics parameter of unlabeled ligands [3,4,5]. However, the Motulsky and Mahan analysis to estimate kinetic binding parameters for unlabeled ligands in these competitive binding assays was found to be sensitive to the used labeled probe [6]. Alternatively, functional assays can be employed to estimate the duration of receptor occupancy by antagonists [7,8]. In contrast to binding assays that are routinely performed with cell homogenates or isolated membranes, functional assays are performed on intact living cells and, consequently also account for dynamic cellular processes such as effector pre-coupling, receptor reserve and receptor regulation. Fast and/or real time cellular responses such as Ca^2+^ influx, cyclic adenosine-monophosphate (cAMP) production and cellular impedance-based assays, have recently been used to measure the recovery of receptor responsiveness as measure of pre-bound antagonist dissociation upon washout of unbound antagonist [3,7,9,10,11,12]. Indeed, recovery time of Gα_q_-coupled histamine H_1_ receptor (H_1_R) responsiveness in a Ca^2+^ mobilization and label free dynamic mass redistribution (DMR) assay after antagonist washout highly correlated to residence times of these antihistamines, as determined in competitive radioligand binding assays [7]. Moreover, response recovery determined utilizing washout experiments corresponds well to the in vivo duration of neurokinin 1 receptor antagonism [12]. Alternatively, estimation of duration of antagonism can be monitored in real-time, utilizing a BRET-based β-arrestin 2 recruitment assay, following co-addition of agonist and antagonist resulting in overshoot-patterns similar to competition association binding assays [13]. In addition, a BRET sensor that monitors cAMP accumulation could effectively determine receptor recovery time (RecT) after washout of pre-incubated antagonist and RecT correlated to residence times from competition binding assays [3]. However, the delayed response of this biosensor upon agonist stimulation of the Gα_i_-coupled receptor hampers quantification of kinetic parameters for relatively fast dissociating ligands.

In this study, we employed a BRET-based assay to measure human histamine H_3_ receptor (hH_3_R)-induced Gα_i2_-protein activation by measuring the very rapid recruitment of Venus-Gβ_1_γ_2_ to a membrane-associated-C-terminal fragment of G protein-coupled receptor kinase 3 (i.e., masGRK3ct-Nluc) that scavenges released Gβγ complexes with high affinity [14,15].

The G protein-coupled receptor (GPCR) H_3_R is a Gα_i_-coupled receptor that functions as pre-synaptic auto-and heteroreceptor and thereby regulates the release of histamine and various other neurotransmitters in the brain [16]. Due to this prominent role in the central nervous system the H_3_R has been associated with a variety of neuropsychiatric disorders such as Parkinson’s disease, epilepsy, learning and sleeping disorders. Although several compounds targeting this receptor have entered clinical trials for different indications, often they are withdrawn in early stages of clinical trials because they are either inefficacious or induce side effects like insomnia [17,18,19]. Many compounds already fail in pre-clinical stages, e.g., ABT-239 due to hERG mediated cardiac toxicity and imidazole containing ligands, like iodophenpropit, due to poor penetration of the blood brain barrier and interference with CYP enzymes. A promising drug candidate as PF03654746 has been tested for several indications (a.o. attention deficit hyperactivity disorder (ADHD), Alzheimer’s disease, allergic rhinitis and schizophrenia), but was discontinued without disclosure of results [20]. Yet, recently pitolisant (Wakix^®^) has been approved [21] by the European Medicines Agency in 2016 for the treatment of narcolepsy to improve wakefulness in patients suffer from excessive daytime sleepiness [22]. In the US, pitolisant has received Breakthrough Therapy and Fast Track designations from the Food and Drug Administration and approval to market this new medication in the United States in 2019 has been requested [23].

Ligands with a short duration of action might be beneficial for the H_3_R to minimize the side effects associated with prolonged inhibition of H_3_R signaling due to its prominent role in neurotransmission. H_3_R antagonists PF03654746, pitolisant and iodophenpropit were assessed for their duration of H_3_R antagonism in a newly developed functional assay format, using living cells. Differences in the recovery of receptor responsiveness and pre-bound antagonists re-equilibration could be observed between the tested H_3_R antagonists.

## 2. Results

### 2.1. BRET between Venus-Gβ_1_γ_2_ and masGRK3ct-Nluc to Measure hH_3_R-Induced Gα_i2_ Activation

**Figure 1:**
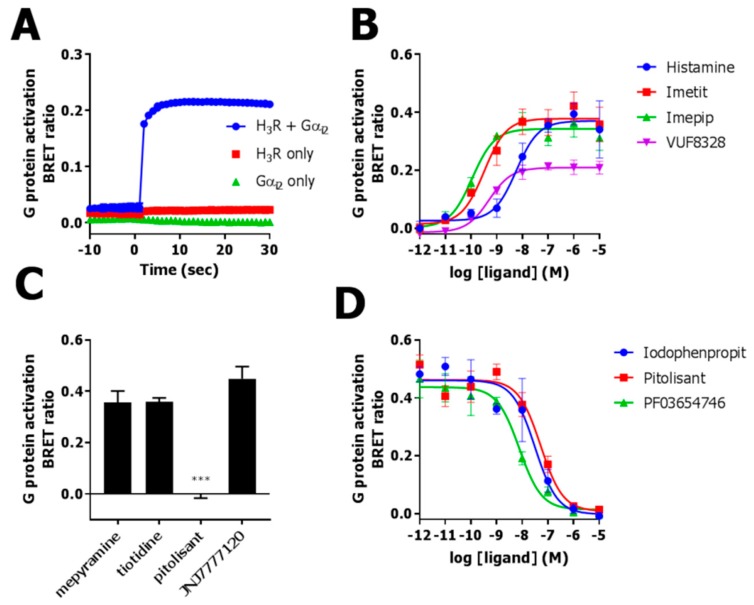
Human H_3_R-mediated Gα_i2_ activation in HEK293T cells measured as bioluminescence resonance energy transfer (BRET) ratio between Venus-Gβ_1_γ_2_ and masGRK3ct-Nluc. (**A**) Real time BRET ratio in HEK293T cells expressing hH_3_R and/or Gα_i2_ upon stimulation with 1 µM histamine at t = 0. (**B**) Concentration-dependent increase in BRET ratio in cells co-expressing hH_3_R and Gα_i2_ upon stimulation with H_3_R agonists. (**C**) BRET ratio in cells co-expressing hH_3_R and Gα_i2_ upon stimulation with 1 µM histamine in the presence of 10 µM histamine receptor subtype-selective antagonists. (**D**) Decrease in histamine-induced (1 µM) BRET ratio in HEK293T cells co-expressing Gα_i2_ in the presence of increasing concentrations H_3_R antagonists. Representative graphs of 3 experiments performed in triplicate are shown and data are mean ± SD. BRET ratio was measured after 10 min incubation (**B**–**D**). BRET ratios are corrected for vehicle. Significance was determined by a Student’s t-test *** (*p* < 0.001).

Heterotrimeric G protein activation by GPCRs can be monitored as the release of Venus-tagged Gβ_1_γ_2_ subunits and their subsequent recruitment toward the membrane-associated Gβγ-scavenger masGRK3ct-Nluc, which increases the BRET signal [14]. Stimulation of adherent HEK293T cells that were co-transfected with Venus-Gβ_1_γ_2_, masGRK3ct-Nluc, hH_3_R and Gα_i2_ cDNA with 1 µM histamine induced a rapid increase in BRET between Venus-Gβ_1_γ_2_ and masGRK3ct-Nluc (Figure 1A), whereas no significant BRET change was observed in cells that were not co-transfected with Gα_i2_ cDNA (*p* = 0.5193, one-way ANOVA). This confirms previous observations that co-expression of exogenous Gα proteins is required for appropriate localization of Venus-Gβ_1_γ_2_ [14]. Histamine did also not change BRET between Venus-Gβ_1_γ_2_ and masGRK3ct-Nluc in cells that were not transfected with hH_3_R cDNA (*p* = 0.8884, one-way ANOVA), demonstrating that the observed Gβ_1_γ_2_ release is indeed mediated via the hH_3_R (Figure 1A).

The known H_3_R agonists histamine, imetit, immepip and VUF8328 all induced a concentration-dependent increase in BRET ratio between Venus-Gβ_1_γ_2_ and masGRK3ct-Nluc in cells that co-express hH_3_R and Gα_i2_ (Figure 1B) with potencies (pEC_50_) and intrinsic activities (α) that are comparable to those reported in a cAMP-responsive element (CRE)-driven reporter gene assay or [^3^H]-cAMP accumulation assay (Table 1) [24,25]. Histamine, imetit, and immepip acted as full agonist in the Gβ_1_γ_2_ release assays, whereas VUF8328 acted as partial agonist (Figure 1B).

The H_3_R antagonist pitolisant (10 µM) fully inhibited Venus-Gβ_1_γ_2_ recruitment to masGRK3ct-Nluc in response to 1 µM histamine, while the H_1_R, H_2_R and H_4_R antagonists (10 µM) mepyramine, tiotidine and JNJ7777120, respectively, were ineffective, confirming that the observed BRET change is indeed hH_3_R dependent (Figure 1C). Moreover, the H_3_R antagonists iodophenpropit, PF03654746, and pitolisant, inhibited histamine-induced Gβ_1_γ_2_ release in a concentration-dependent manner and yielding pK_B_ values that are comparable to their pK_i_ values in a previously reported NanoBRET-based competition binding assay on intact cells that express Nluc-hH_3_R (Figure 1D and Table 2) [3]. 

### 2.2. Duration of Functional hH_3_R Antagonism

**Figure 2:**
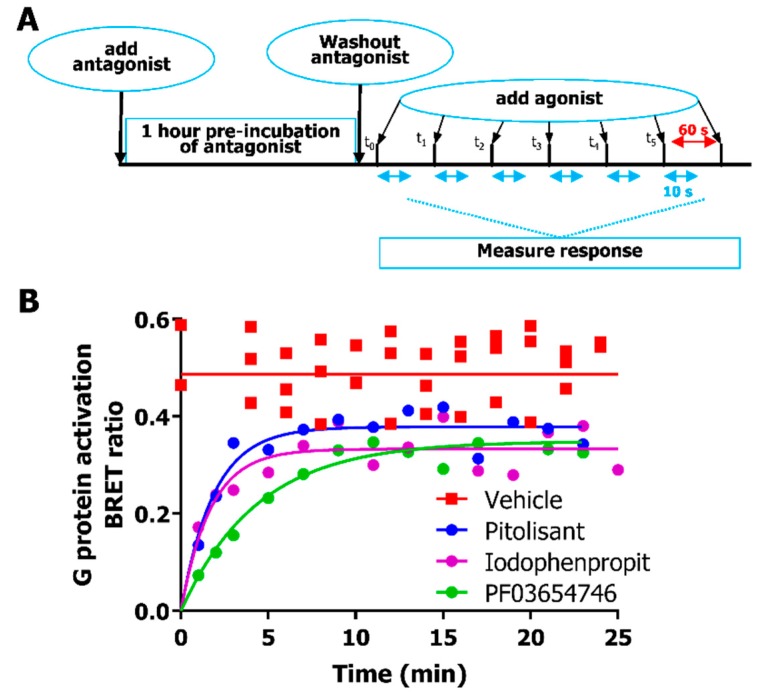
Recovery of hH_3_R-mediated Gα_i2_ activation measured in time as BRET ratio between Venus-Gβγ and masGRK3-Nluc after inhibition with antagonists was monitored for 10 s after agonist addition with 60 s time intervals. (**A**) hH_3_R-expressing HEK293T cells were pre-incubated for 1 h with antagonist (10 × *IC*_50_ concentration) followed by rapid washout of unbound antagonist and stimulation with 10 µM imetit after different incubation times. (**B**) Recovery rate of hH_3_R responsiveness after washout of pre-bound iodophenpropit, pitolisant and PF03654746BRET ratio’s plotted are calculated 10 s after agonist stimulation. Representative graph of 3 independent experiments performed in singular is shown.

The very short time span to achieve maximal Venus-Gβ_1_γ_2_ recruitment to masGRK3ct-Nluc upon agonist H_3_R stimulation allows the detection of duration of antagonism by measuring the recovery of hH_3_R responsiveness to agonist stimulation over time. To this end, hH_3_R was blocked by pre-incubating the cells with antagonist (10 × *IC*_50_ concentration) for 1 h, followed by the removal of unbound antagonist to initiate dissociation from the hH_3_R. Cells were then stimulated with a saturating concentration of imetit (10 µM) after different time intervals and BRET changes between Venus-Gβ_1_γ_2_ and masGRK3ct-Nluc were measured directly and BRET ratio 10 s after agonist stimulation were plotted (Figure 2A,B). The imetit-induced response recovered to a similar steady-state level upon washout of all three antagonists, which was slightly lower as compared with control cells that were pre-incubated with vehicle (Figure 2B). The hH_3_R recovery time was comparable upon washout of iodophenpropit and pitolisant, while hH_3_R recovery was 2 to 2.5-fold slower for PF03654746 (Table 2).

### 2.3. Re-Equilibration Time Determines Whether hH_3_R Antagonism is Insurmountable or Surmountable

**Figure 3:**
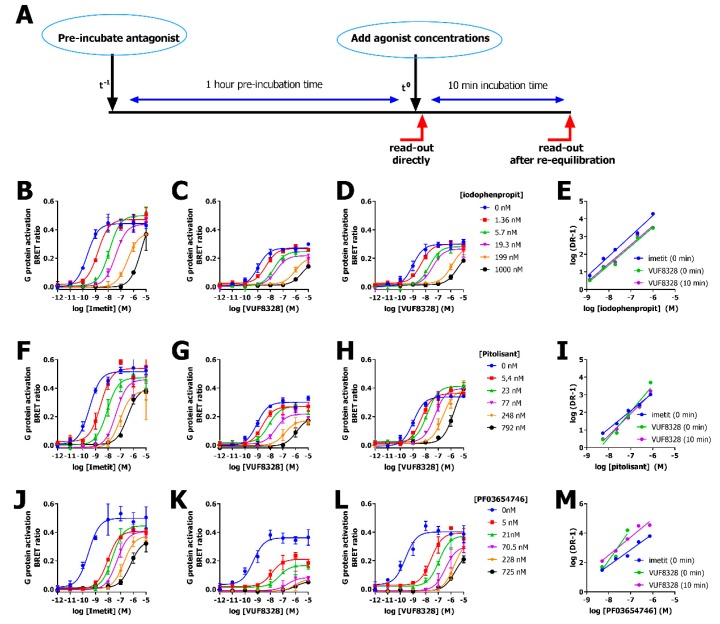
Concentration response curves of imetit in the presence of increasing concentrations of antagonists iodophenpropit (**B**–**D**), pitolisant (**F**–**H**) or PF03654746 (**J**–**L**) measured as BRET ratio between Venus-Gβ_1_γ_2_ and masGRK3ct-Nluc in HEK293T cells co-expressing hH_3_R and Gα_i2_. (**A**) Cells were pre-incubated with antagonists for 1 h prior to stimulation with imetit (**B**,**F**,**J**) or VUF8328 (**C**,**D**,**G**,**H**,**K**,**L**) and BRET ratios were measured immediately (**B**,**C**,**F**,**G**,**J**,**K**) or 10 min (**D**,**H**,**L**) after agonist stimulation. Schild-plots for iodophenpropit (**E**), pitolisant (**I**), and PF03654746 (**M**) contain three regression-lines for the three different DRC panels. Representative graphs of 3 experiments performed in duplicate are shown and data are mean ± SD.

Receptor-induced Venus-Gβ_1_γ_2_ recruitment to masGRK3ct-Nluc can be detected after different time intervals following stimulation with agonist, which offers the opportunity to evaluate the re-equilibration time of pre-bound antagonist within the same agonist-induced response. Cells were pre-incubated for 1 h with multiple concentrations of antagonist and subsequently stimulated with increasing concentrations agonist and BRET ratio was calculated either immediately after agonist addition or after 10 min re-equilibration (Figure 3A).

Pitolisant and PF03654746 acted as insurmountable antagonists if hH_3_R-mediated Venus-Gβ_1_γ_2_ release was measured directly following stimulation with full agonist imetit or partial agonist VUF8328, as revealed by the reduced maximum and rightward-shift of the concentration-response curves (Figure 3F,G,J,K). However, allowing pre-bound antagonists to re-equilibrate with VUF8328 for 10 min before measuring receptor response resulted in surmountable and near-surmountable antagonism for pitolisant and PF03654746, respectively (Figure 3H,L). In contrast, iodophenpropit displayed surmountable antagonism by shifting the imetit and VUF8328 concentration-response curves parallel rightward without attenuating the maximal response if measured either directly or 10 min after agonist stimulation (Figure 3B,C). The observed difference in re-equilibration time confirmed the slightly longer receptor recovery time (RecT) for PF03654746 in comparison with iodophenpropit and pitolisant. Double logarithmic Schild plot analysis revealed a linear relationship between the equiactive dose ratios (DR-1) and antagonist concentration for all three antagonists (Figure 3E,I,M). The Schild regression slopes were not significantly different from unity for iodophenpropit-mediated inhibition of imetit- and VUF8328-induced hH_3_R signaling, indicating that iodophenpropit acted as competitive antagonist (Figure 3D). In contrast, slopes of Schild-regression for PF03654746 and pitolisant were larger than 1 (slope_(0 min)_: 1.5 ± 0.2 and slope_(0 min)_: 1.4 ± 0.0, respectively) if hH_3_R signaling is measured directly upon VUF8328 stimulation, indicating non-competitive antagonism due to insufficient re-equilibration time [26,27]. After 10 min re-equilibration with VUF8328, the Schild regression slopes for both pitolisant and PF03654746 were not significantly different from unity (*p* = 0.15 and 0.18 for Pitolisant and PF03654746, respectively, Student’s t-test) (Table 2, Figure 3I,M). The fitted pA_2_ values for all three antagonists were comparable between the responses that were measured directly or after 10 min upon agonist addition, and in the same order as their pK_B_ and pK_i_ values (Table 2). 

## 3. Discussion

The preferred residence time and consequent duration of drug action might be highly target dependent [28,29]. For example, antihistamines should preferentially occupy the H_1_R for a long duration to prevent an allergic reaction in the case of hay fever. On the other hand, prolonged blockade of the H_3_R leads to undesired on-target side effect insomnia, and consequently relatively short residence time antagonists might be preferred for this receptor [19,30]. Early withdrawal of drug candidates from clinical trials might be reduced if duration of drug action is already taken into account at a very early stage in the drug discovery process. Recently, we evaluated the duration of functional H_3_R antagonism using the BRET-based cAMP biosensor CAMYEL, and indeed observed a correlation between the target recovery time upon antagonist washout and target residence time in competitive association binding assays [3]. However, detection of H_3_R-mediated reduction in cAMP levels using this CAMYEL biosensor was delayed in time upon agonist stimulation, due to the required activation of adenylyl cyclase by forskolin. Therefore, prompt detection of Gα_i_ protein activation via Gβ_1_γ_2_ release rather than the downstream effect on cAMP levels might be more suitable to evaluate antagonist binding kinetics. Indeed, fast recovery (<20 min) of H_3_R-induced Gβ_1_γ_2_ release to steady-state level in response to agonist stimulation upon washout of pre-bound iodophenpropit, pitolisant, and PF03654746 was observed. However, steady-state levels were slightly lower as compared with the agonist-induced response in cells that were pre-treated with vehicle, which might be the consequence of re-binding of antagonist that has dissociated from the receptor or partitioned in the cell membrane after washout of unbound antagonist [31]. In contrast, a full recovery of the hH_3_R responsiveness was previously observed after >30 min following washout of pre-bound pitolisant in the CAMYEL biosensor assay [3]. The recovery of agonist-induced Gβ_1_γ_2_ release was approximately two-fold faster upon iodophenpropit and pitolisant washout as compared with PF03654746, suggesting that the latter antagonist has a longer H_3_R residence time. Competitive association binding on intact HEK293T cells, however, previously indicated that pitolisant and PF03654746 have a comparable residence time of approximately 15 min [3,32]. The incomplete re-equilibration of pre-bound PF03654746 within the 75 s time-course of a transient Ca^2+^ peak response leads to a reduced maximum of the R-α-methyl-histamine concentration response curves as unoccupied receptors are not available for agonist stimulation [32]. Similar insurmountable antagonism by pre-bound PF03654746 was observed when BRET between Venus-Gβ_1_γ_2_ and masGRK3ct-Nluc was measured within the same short time frame after agonist-stimulation, whereas PF03654746 acted as (nearly) surmountable antagonist (i.e., parallel dextral shift of agonist response curve without attenuation of maximum response) if this response was measured after a 10 min re-equilibration period. Hence, the required re-equilibration time for a pre-bound insurmountable antagonist (i.e., parallel dextral shift combined with depression of maximum agonist response) to become a surmountable antagonist (i.e., parallel dextral shift of agonist response curve without attenuation of maximum response) is indicative for the target residence time of the antagonist [27,33]. Indeed, short residence time antagonist iodophenpropit caused a parallel rightward shift of these curves directly upon agonist stimulation, whereas longer residence time antagonists pitolisant and PF03654746 decreased the maximum response directly following VUF8328 stimulation but not after 10 min incubation period, suggesting that these pre-bound antagonists re-equilibrate within this time-frame.

In conclusion, knowledge on how long an antagonist occupies its target is considered to be an important parameter in drug discovery. The very fast kinetics of the Gβ_1_γ_2_ release response makes this BRET-based assay a valuable tool to measure duration of target occupancy by washout of pre-bound antagonist and detection of the recovery of agonist responsiveness. Moreover, in contrast to frequently used, but very transient calcium responses, this Gβ_1_γ_2_ release can be measured after increasing time intervals following agonist stimulation, which allows detection of antagonist re-equilibration by measuring Schild analysis experiments in time. The only marketed H_3_R antagonist pitolisant (Wakix^®^) showed relative fast dissociation kinetics in both Gβ_1_γ_2_ release and hemi-equilibrium Ca^2+^ assays, suggesting that short-residence time antagonists might display best in vivo efficacy to therapeutically target hH_3_R [32].

## 4. Materials and Methods

### 4.1. Materials

PF03654746 was bought from Axon Medchem (Groningen, The Netherlands) and pitolisant was obtained from Griffin Discoveries (Amsterdam, The Netherlands). All other compounds were synthesized in-house as previously reported [34,35,36]. Human embryonic kidney 293T cells (HEK293T cells) were obtained from ATCC (Manassas, VA, USA). Fetal bovine serum (FBS) was obtained from Bodinco (Alkmaar, The Netherlands). Penicillin and streptomycin were purchased from GE healthcare (Uppsula, Sweden). Dulbecco’s Modified Eagles Medium (DMEM), trypsin-EDTA and Hanks’ Balanced Salt Solution (HBSS) were obtained from Gibco (Thermo Fisher Scientific, Waltham, MA, USA). Nano-Glo^®^ (3.2 µL/mL) was obtained from Promega (Madison, WI, USA). Plasmids encoding for Venus155-239-Gβ_1_, Venus1-155-Gγ_2_ and masGRK3ct-Nluc were kindly provided by Dr. N. Lambert (Georgia Health Sciences University, Augusta, GA, USA). PcDNA3.1+ plasmid encoding human Gα_i2_ subunit was bought from cDNA Resource Center (Bloomsberg, PA, USA). Human H_3_R (Genbank accession no. AF140538) in pcDEF3 was previously described [37]. All other chemicals were of analytical grade and obtained from standard commercial sources.

### 4.2. Cell Culture and Transfection

Human embryonic kidney 293T cells (HEK293T cells) were cultured in DMEM supplemented with 10% FBS, penicillin (100 µg/mL) and streptomycin (50 µg/mL) at 37 °C with 5% CO_2_. Cells were transiently transfected in a 10 cm^2^ dish with plasmids coding for Venus155-239-Gβ_1_ (0.4 µg), Venus1-155-Gγ_2_ (0.4 µg), masGRK3ct-Nluc (0.4 µg), hH_3_R (0.4 µg), Gα_i2_-protein (1.2 µg), and empty pcDEF3 (2.2 µg) using 20 µg 25 kDa linear polyethylenimine (Polysciences Inc, Warrington, PA, USA), as previously described [3]. The next day, 50,000 cells/well were transferred to a poly-L-lysine coated black 96 well plates (Greiner Bio-one GmbH, Frickenhausen, Germany) and grown for an additional 24 h. 

### 4.3. BRET between Venus-Gβ_1_γ_2_ and masGRK3ct-Nluc to Measure H_3_R-Induced Gα_i2_ Activation

Agonist-induced BRET between Venus-Gβ_1_γ_2_ and masGRK3ct-Nluc was measured on adherent HEK293T cells co-expressing hH_3_R and Gα_i2_ in the presence of NanoGlo^®^ (3.2 µL/mL) using a Mithras LB940 multimode microplate reader (Berthold, Germany). Cells were stimulated with histamine in the absence and presence of antagonist in HBSS buffer, and BRET (540–40 nm) and luminescence (480–20 nm) signals were monitored either immediately or after 10 min at 25 °C. Recovery of hH_3_R responsiveness was measured following 1 h pre-incubation with antagonist (10 × *IC*_50_ concentration) and two wash steps with HBSS to remove unbound antagonist. Next, cells were incubated in HBSS and stimulated with 10 μM imetit after various incubation times. BRET between Venus-G_1_βγ_2_ and masGRK3ct-Nluc was immediately measured upon imetit stimulation and BRET ratio after 10 s were plotted. Re-equilibration of pre-bound antagonists was measured upon pre-incubation of the cells with increasing concentrations of antagonist for one hour prior to the addition of increasing concentrations imetit or VUF8328 and BRET ratio was calculated either immediately after agonist addition (0–3 min) or after 10 min re-equilibration.

### 4.4. Data Analysis

Ligand-induced BRET changes were calculated by dividing emission at 540–40 nm (Venus) by emission at 480–20 nm (Nluc) and baseline-corrected by subtracting BRET of vehicle. Representative graphs of at least three independent experiments are shown and analyzed by linear or non-linear regression using Prism 7.03 (GraphPad Software, San Diego, CA, USA). Agonist and antagonist concentration-response curves were fitted to three-parameter response models to obtain *EC*_50_ and *IC*_50_ values, respectively. The equilibrium dissociation constant of antagonist (*K*_B_) for the hH_3_R is calculated from *IC*_50_ values using:

(1) $$K_{B}\;=\;\frac{IC_{50}}{\left(\frac{\left[A\right]}{EC_{50}}+1\right)}$$

where [*A*] and *EC*_50_ are the histamine concentration (10 µM) and potency, respectively. For receptor recovery the vehicle corrected BRET ratio at 10 s after agonist addition was plotted over time.

Recovery of receptor responsiveness (*Y*) was analyzed as function of time by a one-phase association model:

(2) $$Y=Y_{0}+\left(Y_{max}-Y_{0}\right)\times \left(1-e^{-k_{rec}\times t}\right)$$

where *Y*_0_ is the hH_3_R response induced by 10 µM imetit in the presence of (10 × *IC*_50_ concentration) antagonist, *Y_max_* is the imetit-induced response upon reaching steady-state and *k_rec_* is the receptor-recovery rate in min^−1^. The receptor recovery time (RecT) was calculated as the reciprocal of the *k_rec_*.

Equiactive agonist concentrations for Schild analysis were determined from concentration response curves at 10 % max agonist response (vide supra) in the absence and presence of increasing antagonist concentrations. Dose ratios (*DR*) were calculated by dividing the equiactive agonist concentration in the presence of antagonist by the equiactive agonist concentration in the absence of antagonist. *DR* minus 1 was plotted as function of antagonist concentration ([*B*]) in a double logarithmic graph and pA_2_ and slope values were determined by linear regression:

(3) $$\log\left(DR-1\right)=\log\left[B\right]+pA_{2}$$

Statistical analysis was performed using Graphpad Prism 7.03.

## Figures and Tables

**Table 1:** Potencies, intrinsic activities (α) of ligands in hH_3_R-mediated Gα_i2_ activation in HEK293T cells measured as recruitment of Venus-Gβ_1_γ_2_ to masGRK3ct-Nluc. Data shown are mean ± S.E.M. of three experiments performed in triplicate.

Compound	pEC50 ± S.E.M.	α	pKi ^a^
Histamine	8.3 ± 0.0	1.0 ± 0.0	6.7
Imetit	9.6 ± 0.1	1.1 ± 0.1	8.6
immepip	9.9 ± 0.1	1.0 ± 0.1	8.8
VUF8328	9.4 ± 0.1	0.7 ± 0.2	8.5

^a^ pKi values obtained from previously reported competition binding studies [3,24].

**Table 2:** Equilibrium dissociation constants (pK_B_), pA_2_-values and slopes from Schild-regression and recovery time of ligands in hH_3_R-mediated Gα_i2_ activation in HEK293T cells measured as recruitment of Venus-Gβ_1_γ_2_ to masGRK3ct-Nluc. Data shown are mean ± S.E.M. of three experiments performed in triplicate except for RecT, which was measured in singlo.

Compound	pK_B_^a^	pK_i_^b^	Imetit _(0 min)_		VUF8328 _(0 min)_		VUF8328 _(10 min)_		RecT ^c^ (min)
			pA_2_	Slope	pA_2_	Slope	pA_2_	Slope	
Iodophenpropit	9.5 ± 0.1	9.4	9.6 ± 0.0	1.1 ± 0.0	9.3 ± 0.2	1.1 ± 0.1	9.5 ± 0.3	1.1 ± 0.1	1.4 ± 0.2
Pitolisant	9.1 ± 0.0	8.9	8.9 ± 0.1	1.1 ± 0.0	8.4 ± 0.0	1.4 ± 0.0	8.7 ± 0.2	1.1 ± 0.1	1.7 ± 0.3
PF03,654,746	9.9 ± 0.1	9.3	9.8 ± 0.1	1.0 ± 0.1	9.3 ± 0.2	1.5 ± 0.2	9.6 ± 0.2	1.2 ± 0.1	3.6 ± 0.3

^a^ pK_B_ values are calculated from *IC*_50_ values, ^b^ pK_i_ values obtained from previously reported competition binding studies [3], ^c^ RecT values are calculated as 1/k_rec_.

## Abbreviations

α	Intrinsic activity
ADHD	Attention deficit hyperactivity disorder
ANOVA	Analysis of variance
BRET	Bioluminescence resonance energy transfer
cAMP	cyclic Adenosine-MonoPhosphate
DMEM	Dulbecco’s Modified Eagles Medium
DMR	Dynamic mass redistribution
*DR*	Dose ratio
GPCR	G protein-coupled receptor
FBS	Fetal bovine serum
H_1_R	Histamine H_1_ receptor
H_3_R	Histamine H_3_ receptor
HEK	Human embryonic kidney
hERG	Human ether-a-go-go related gene
hH_3_R	Human H_3_R
*IC*_50_	Concentration inhibitor needed to reduce response by 50%
*k*_off_	Dissociation rate constant
masGRK3ct	membrane-associated-C-terminal fragment of G protein-coupled receptor kinase 3
Nluc	NanoLuc luciferase
pEC_50_	−log (EC_50_) where EC_50_ is concentration required to obtain half maximal response
pKi	−log K_i_, where Ki is the equilibrium dissociation constant
RecT	Recovery time

## References

[1] Copeland R.A., Pompliano D.L., Meek T.D. (2006). Drug-target residence time and its implications for lead optimization. Nat. Rev. Drug Discov..

[2] Sykes D.A., Stoddart L.A., Kilpatrick L.E., Hill S.J. (2019). Binding kinetics of ligands acting at GPCRs. Mol. Cell. Endocrinol..

[3] Mocking T.A.M., Verweij E.W.E., Vischer H.F., Leurs R. (2018). Homogeneous, Real-Time NanoBRET Binding Assays for the Histamine H3 and H4 Receptors on Living Cells. Mol. Pharmacol..

[4] Bouzo-Lorenzo M., Stoddart L.A., Xia L., IJzerman A.P., Heitman L.H., Briddon S.J., Hill S.J. (2019). A live cell NanoBRET binding assay allows the study of ligand-binding kinetics to the adenosine A3 receptor. Purinergic Signal..

[5] Stoddart L.A., Vernall A.J., Bouzo-Lorenzo M., Bosma R., Kooistra A.J., de Graaf C., Vischer H.F., Leurs R., Briddon S.J., Kellam B. (2018). Development of novel fluorescent histamine H1-receptor antagonists to study ligand-binding kinetics in living cells. Sci. Rep..

[6] Bosma R., Stoddart L.A., Georgi V., Bouzo-Lorenzo M., Bushby N., Inkoom L., Waring M.J., Briddon S.J., Vischer H.F., Sheppard R.J. (2019). Probe dependency in the determination of ligand binding kinetics at a prototypical G protein-coupled receptor. Sci. Rep..

[7] Bosma R., Witt G., Vaas L.A.I., Josimovic I., Gribbon P., Vischer H.F., Gul S., Leurs R. (2017). The target residence time of antihistamines determines their antagonism of the G protein-coupled histamine H1 receptor. Front. Pharmacol..

[8] Bosma R., van den Bor J., Vischer H.F., Labeaga L., Leurs R. (2018). The long duration of action of the second generation antihistamine bilastine coincides with its long residence time at the histamine H1 receptor. Eur. J. Pharmacol..

[9] Nederpelt I., Kuzikov M., De Witte W.E.A., Schnider P., Tuijt B., Gul S., IJzerman A.P., De Lange E.C.M., Heitman L.H. (2017). From receptor binding kinetics to signal transduction; a missing link in predicting in vivo drug-action. Sci. Rep..

[10] Doornbos M.L.J., Cid J.M., Haubrich J., Nunes A., Van De Sande J.W., Vermond S.C., Mulder-Krieger T., Trabanco A.A., Ahnaou A., Drinkenburg W.H. (2017). Discovery and Kinetic Profiling of 7-Aryl-1,2,4-triazolo[4,3-a]pyridines: Positive Allosteric Modulators of the Metabotropic Glutamate Receptor 2. J. Med. Chem..

[11] Slack R.J., Russell L.J., Hall D.A., Luttmann M.A., Ford A.J., Saunders K.A., Hodgson S.T., Connor H.E., Browning C., Clark K.L. (2011). Pharmacological characterization of GSK1004723, a novel, long-acting antagonist at histamine H 1 and H 3 receptors. Br. J. Pharmacol..

[12] Lindström E., von Mentzer B., Påhlman I., Ahlstedt I., Uvebrant A., Kristensson E., Martinsson R., Novén A., de Verdier J., Vauquelin G. (2007). Neurokinin 1 Receptor Antagonists: Correlation between in Vitro Receptor Interaction and in Vivo Efficacy. J. Pharmacol. Exp. Ther..

[13] Bosma R., Moritani R., Leurs R., Vischer H.F. (2016). BRET-based β-arrestin2 recruitment to the histamine H1 receptor for investigating antihistamine binding kinetics. Pharmacol. Res..

[14] Masuho I., Ostrovskaya O., Kramer G.M., Jones C.D., Xie K., Martemyanov K.A. (2015). Distinct profiles of functional discrimination among G proteins determine the actions of G protein-coupled receptors. Sci. Signal..

[15] Hollins B., Kuravi S., Digby G.J., Lambert N.A. (2009). The c-terminus of GRK3 indicates rapid dissociation of G protein heterotrimers. Cell. Signal..

[16] Panula P., Chazot P.L., Cowart M., Gutzmer R., Leurs R., Liu W.L.S., Stark H., Thurmond R.L., Haas H.L. (2015). International Union of Basic and Clinical Pharmacology. XCVIII. Histamine Receptors. Pharmacol. Rev..

[17] Ghamari N., Zarei O., Arias-Montaño J.A., Reiner D., Dastmalchi S., Stark H., Hamzeh-Mivehroud M. (2019). Histamine H3 receptor antagonists/inverse agonists: Where do they go?. Pharmacol. Ther..

[18] Kuhne S., Wijtmans M., Lim H.D., Leurs R., de Esch I.J. (2011). Several down, a few to go: Histamine H3 receptor ligands making the final push towards the market?. Expert Opin. Investig. Drugs.

[19] Sadek B., Łażewska D., Hagenow S., Kieć-Kononowicz K., Stark H., Blandina P., Passani M.B. (2016). Histamine H3R Antagonists: From Scaffold Hopping to Clinical Candidates. Histamine Receptors: Preclinical and Clinical Aspects.

[20] Stokes J.R., Romero F.A., Allan R.J., Phillips P.G., Hackman F., Misfeldt J., Casale T.B. (2012). The effects of an H 3 receptor antagonist (PF-03654746) with fexofenadine on reducing allergic rhinitis symptoms. J. Allergy Clin. Immunol..

[21] Kollb-Sielecka M., Demolis P., Emmerich J., Markey G., Salmonson T., Haas M. (2017). The European Medicines Agency review of pitolisant for treatment of narcolepsy: Summary of the scientific assessment by the Committee for Medicinal Products for Human Use. Sleep Med..

[22] Romigi A., Vitrani G., Lo Giudice T., Centonze D., Franco V. (2018). Profile of pitolisant in the management of narcolepsy: Design, development, and place in therapy. Drug Des. Dev. Ther..

[23] Bioprojet: Pitolisant Progresses Towards The U.S. Market. http://www.bioprojet.com/en/article/harmony-fda-2/.

[24] Wieland K., Bongers G., Yamamoto Y., Hashimoto T., Yamatodani A., Menge W.M.B.P., Timmerman H., Lovenberg T.W., Leurs R. (2001). Constitutive Activity of Histamine H3 Receptors Stably Expressed in SK-N-MC Cells: Display of Agonism and Inverse Agonism by H3 Antagonists. J. Pharmacol. Exp. Ther..

[25] Lim H.D., van Rijn R.M., Ling P., Bakker R.A., Thurmond R.L., Leurs R. (2005). Evaluation of histamine H1-, H2-, and H3-receptor ligands at the human histamine H4 receptor: Identification of 4-methylhistamine as the first potent and selective H4 receptor agonist. J. Pharmacol. Exp. Ther..

[26] Vauquelin G., Van Liefde I., Birzbier B.B., Vanderheyden P.M.L. (2002). New insights in insurmountable antagonism. Fundam. Clin. Pharmacol..

[27] Kenakin T., Jenkinson S., Watson C. (2006). Determining the potency and molecular mechanism of action of insurmountable antagonists. J. Pharmacol. Exp. Ther..

[28] Schuetz D.A., De Witte W.E.A., Wong Y.C., Knasmueller B., Richter L., Kokh D.B., Sadiq S.K., Bosma R., Nederpelt I., Heitman L.H. (2017). Kinetics for Drug Discovery: An industry-driven effort to target drug residence time. Drug Discov. Today.

[29] Vanderheyden P.M.L., Benachour N. (2017). Influence of the cellular environment on ligand binding kinetics at membrane-bound targets. Bioorg. Med. Chem. Lett..

[30] Wijtmans M., Leurs R., de Esch I. (2007). Histamine H3 receptor ligands break ground in a remarkable plethora of therapeutic areas. Expert Opin. Investig. Drugs.

[31] Sykes D.A., Parry C., Reilly J., Wright P., Fairhurst R.A., Charlton S.J. (2014). Observed Drug-Receptor Association Rates Are Governed by Membrane Affinity: The Importance of Establishing “Micro-Pharmacokinetic/Pharmacodynamic Relationships” at the 2-Adrenoceptor. Mol. Pharmacol..

[32] Riddy D.M., Cook A.E., Shackleford D.M., Pierce T.L., Mocaer E., Mannoury la Cour C., Sors A., Charman W.N., Summers R.J., Sexton P.M. (2019). Drug-receptor kinetics and sigma-1 receptor affinity differentiate clinically evaluated histamine H3 receptor antagonists. Neuropharmacology.

[33] Mould R., Brown J., Marshall F.H., Langmead C.J. (2014). Binding kinetics differentiates functional antagonism of orexin-2 receptor ligands. Br. J. Pharmacol..

[34] Van der Goot H., Schepers M., Sterk G., Timmerman H. (1992). Isothiourea analogues of histamine as potent agonists or antagonists of the histamine H3-receptor. Eur. J. Med. Chem..

[35] Jansen F.P., Wu T.S., Voss H.P., Steinbusch H.W.M., Vollinga R.C., Rademaker B., Bast A., Timmerman H. (1994). Characterization of the binding of the first selective radiolabeled histamine H3-receptor antagonist, [125I]-iodophenpropit, to rat brain. Br. J. Pharmacol..

[36] Vollinga R.C., de Koning J.P., Jansen F.P., Leurs R., Menge W.M.P.B., Timmerman H. (1994). A New Potent and Selective Histamine H3 Receptor Agonist, 4-(1H-imidazol-4-ylmethyl)piperidine. J. Med. Chem..

[37] Bongers G., Krueger K.M., Miller T.R., Baranowski J.L., Estvander B.R., Witte D.G., Strakhova M.I., van Meer P., Bakker R.A., Cowart M.D. (2007). An 80-Amino Acid Deletion in the Third Intracellular Loop of a Naturally Occurring Human Histamine H3 Isoform Confers Pharmacological Differences and Constitutive Activity. J. Pharmacol. Exp. Ther..

